# Walking stability in patients with benign paroxysmal positional vertigo: an objective assessment using wearable accelerometers and machine learning

**DOI:** 10.1186/s12984-021-00854-y

**Published:** 2021-03-31

**Authors:** Yuqian Zhang, He Wang, Yifei Yao, Jianren Liu, Xuhong Sun, Dongyun Gu

**Affiliations:** 1 Shanghai Key Laboratory of Orthopaedic Implants, Department of Orthopaedic Surgery, Shanghai Ninth People’s Hospital, Shanghai Jiao Tong University School of Medicine, Shanghai, 200011 People’s Republic of China; 2 School of Biomedical Engineering and Med-X Research Institute, Shanghai Jiao Tong University, Shanghai, 200030 People’s Republic of China; 3 Engineering Research Center of Digital Medicine and Clinical Translation, Ministry of Education of People’s Republic China, Shanghai, 200030 People’s Republic of China; 4 Department of Neurology, Shanghai Ninth People’s Hospital, Shanghai Jiao Tong University School of Medicine, Shanghai, 200011 People’s Republic of China

**Keywords:** Benign paroxysmal positional vertigo, Walking stability, Gait analysis, Wearable sensors, Machine learning model

## Abstract

**Background:**

Benign paroxysmal positional vertigo (BPPV) is one of the most common peripheral vestibular disorders leading to balance difficulties and increased fall risks. This study aims to investigate the walking stability of BPPV patients in clinical settings and propose a machine-learning-based classification method for determining the severity of gait disturbances of BPPV.

**Methods:**

Twenty-seven BPPV outpatients and twenty-seven healthy subjects completed level walking trials at self-preferred speed in clinical settings while wearing two accelerometers on the head and lower trunk, respectively. Temporo-spatial variables and six walking stability related variables [root mean square (RMS), harmonic ratio (HR), gait variability, step/stride regularity, and gait symmetry] derived from the acceleration signals were analyzed. A support vector machine model (SVM) based on the gait variables of BPPV patients were developed to differentiate patients from healthy controls and classify the handicapping effects of dizziness imposed by BPPV.

**Results:**

The results showed that BPPV patients employed a conservative gait and significantly reduced walking stability compared to the healthy controls. Significant different mediolateral HR at the lower trunk and anteroposterior step regularity at the head were found in BPPV patients among mild, moderate, and severe DHI (dizziness handicap inventory) subgroups. SVM classification achieved promising accuracies with area under the curve (AUC) of 0.78, 0.83, 0.85 and 0.96 respectively for differentiating patients from healthy controls and classifying the three stages of DHI subgroups. Study results suggest that the proposed gait analysis that is based on the coupling of wearable accelerometers and machine learning provides an objective approach for assessing gait disturbances and handicapping effects of dizziness imposed by BPPV.

## Introduction

Benign paroxysmal positional vertigo (BPPV) is considered to be the most common peripheral vestibular disorder with a lifetime prevalence of 2.4 % [1]. The vestibular system senses the linear and angular acceleration of the head during movement, and this plays a critical role in stabilizing gaze, head, and trunk during movement in order to maintain balance. Due to the impaired vestibular system in BPPV, patients usually suffer from transient vertigo and nystagmus leading to balance difficulties, increased risk of falls, and generally reduced quality of life [1, 2].

The Dix-Hallpike (DH) test is regarded as the gold standard diagnostic test for BPPV, which is performed by moving the patient position to trigger nystagmus [3]. However, there are some limitations to the DH test. During the DH test, patients need to passively recline their upper body and extend their head and neck into the intense vertigo-provoking position. Further, patients must tolerate at least 30-seconds of head hanging supported only by the hands of an examiner, while withstanding vertigo. This inevitably causes severe fright and discomfort in the patient, thus patients with any cervical spine or neck problem cannot participate in the test [4]. The Dizziness Handicap Inventory (DHI), a 25-item self-assessment scale designed to measure the self-perceived level of handicap associated with the symptom of dizziness, has been proposed to assist in the diagnosis of BPPV and quantify the handicapping effects of dizziness in vestibular disorders [5, 6]. Previous studies have shown that there are significant differences in DHI scores between healthy people and BPPV [5, 7]. However, DHI is based on self-perception of disease and therefor there is still a lack of an objective tool to assess the severity of BPPV disease associate with dizziness handicapping.

Walking is a precision task and highly related to dynamic balance ability, which requires the maintenance of a stable gaze as well as a stable head and trunk movement to avoid falls. However, a stable gait remains a challenge in BPPV due to their impaired vestibular system. Previous studies have evaluated the walking performance of BPPV patients during normal gait and tandem walk, and impaired temporospatial variables were observed in these studies [8–10]. These results could only indicate a conservative gait adopted in BPPV to avoid falls but could not answer why they are still at high risk of falling. Another limitation of previous studies is that the measurement was conducted in laboratory settings and required sophisticated equipment such as 3D motion capture system, which could not truly reflect the gait disturbances during transient vertigo in BPPV patients.

Walking stability during natural walking have been used to quantify the balance ability and disease severity, which can be accessed using wearable sensors without the limitations of a gait laboratory environment [11–13]. The sensor-based measurements of walking stability include acceleration root mean square (RMS) harmonic ratio (HR), gait variability, gait symmetry and gait regularity [14]. Previous studies have found that BPPV patients have impaired abilities in controlling static posture balance in mediolateral and anteroposterior axes [15, 16], thus it may help us to gain insights into the BPPV disease better by analyzing the walking stability in various axes rather than purely studying the temporospatial gait variables. Furthermore, previous studies have found the significant associations between the vestibular dysfunction and the changes of gait and balance, thus offering a possibility to objectively assess the severity of gait disturbances imposed by BPPV disease [17–19].

Therefore, the aim of this study was to quantitatively analyze the walking stability of patients with BPPV using accelerometers in clinical settings, and further to explore a method for the assessment of handicapping effects of dizziness imposed by BPPV. We hypothesized that patients with BPPV would exhibit impaired walking stability compared with healthy controls even if a conservative gait was adopted. We further hypothesized that the impaired gait variables are associated with the DHI scores, and a machine learning-based model may objectively assess the handicapping effects of dizziness imposed by BPPV.

## Materials and methods

### Subjects

Twenty-seven outpatients diagnosed with active, idiopathic unilateral BPPV of the posterior semicircular canal between the ages of 30–70 years [average 56.5 (SD13.1)], and 27 healthy subjects between the ages of 25–70 years [average 56.1 (SD10.8)] were included in this study (Table 1). None of the healthy subjects had any medical history of neurological or orthopaedic conditions. According to the classification of patient’s functional abilities by DHI scores, the BPPV patients were classified into three subgroups: mild stage (DHI = 0–30), moderate stage (DHI = 31–60), and severe stage (DHI = 61–100).

The procedures of this study were approved and mandated by the institutional human research ethics committee of School of Biomedical Engineering, Shanghai Jiao Tong University (protocol number:2018007), and conformed to the 1964 Helsinki Declaration. All subjects were fully informed of the study procedures, possible risks, privacy, and the freedom to withdraw. Informed consent was obtained from all participants.

### Experiment setup

**Fig. 1:**
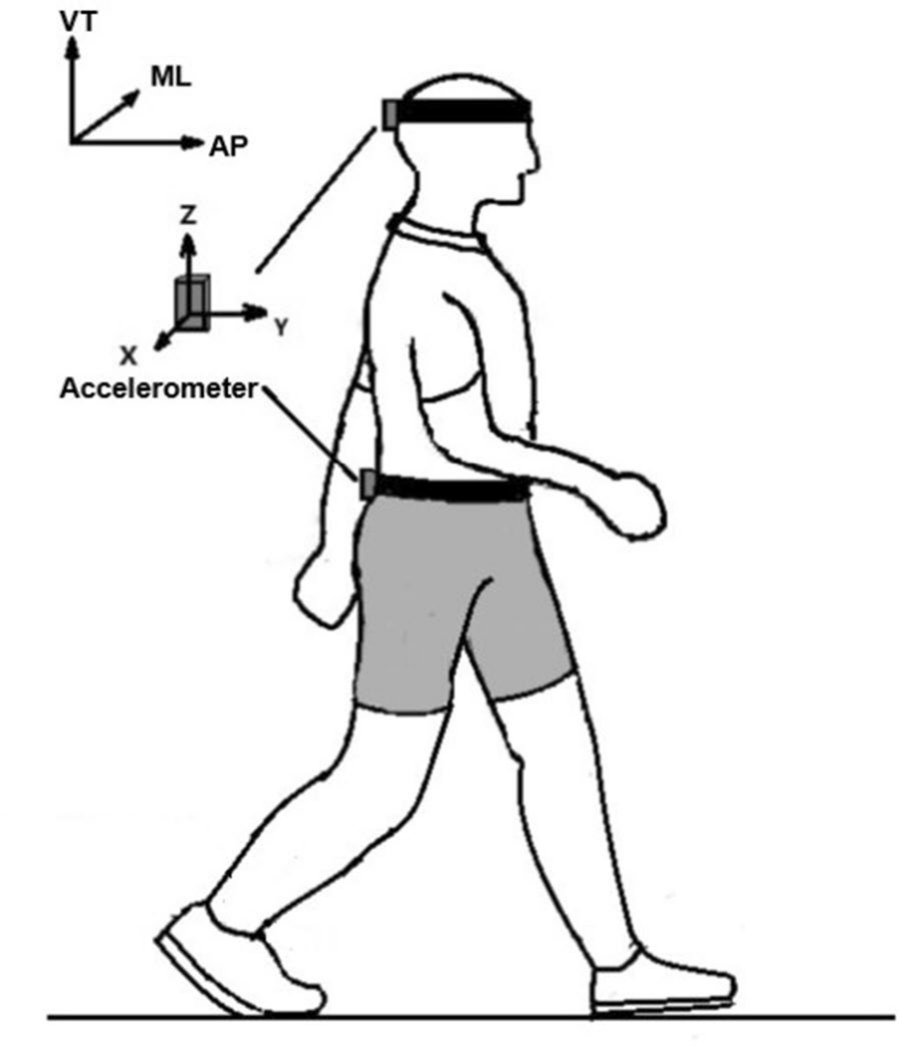
Two accelerometers at the back of head and the third lumbar spinous process(L3), where the X-axis pointed to the right representing the mediolateral (ML) axis, the Y-axis pointed forwards representing the anteroposterior (AP) axis, and the Z-axis pointed to the upwards representing the vertical (VT) axis

Level walking experiments were performed in the outpatient corridor of the neurology department at the Shanghai Ninth People’s Hospital. All subjects were instructed to walk at self-preferred speed along a 20 m walkway, during which their head was not allowed to turn, eyes looking straight ahead, and arms swinging naturally. A Timing Gait System (Brower, Draper, Utah, USA) was used to measure the walking duration of the middle 10 m of steady walk. The trial was defined as invalid if the standard deviation of the walking duration of each subject exceeded 5 %. In this study, 6 valid trials were obtained for each subject. Two accelerometers (Delsys, Inc., Boston, MA, USA) were firmly attached with belt to the back of subject’s head (at the level of frontal lobe) and the third lumbar spinous process (L3, one lumbar vertebra above the midpoint of the bilateral iliac crests), respectively (Fig. 1). Calibration was performed before each walking trail by placing the sensors align with the spine vertically to ensure the vertical acceleration is statically the ± 1 g value. Acceleration signals were captured by Delsys acquisition software (Delsys, Inc., Boston, MA, USA) and recorded at 148 Hz sampling rate in three orthogonal axes (VT, AP, and ML), respectively.

Figure 1 two accelerometers at the back of head and third lumbar spinous process (L3), where the X-axis pointed to the right representing the mediolateral (ML) axis, the Y-axis pointed forwards representing the anteroposterior (AP) axis, and the Z-axis pointed to the upwards representing the vertical (VT) axis.

### Data processing and gait variable calculation

The gravity component was first removed from the raw acceleration data and then filtered with a second-order Butterworth low pass filter with a cutoff frequency of 22 Hz. Five clinically relevant temporospatial variables and six variables reflecting walking stability were selected and calculated in Matlab (2019 a, the MathWorks, Inc., Natick, MA, USA).

#### Temporo‐spatial variables

*Walking speed (m/s)*, walking distance (10 m) divided by the total time duration measured by timing gait system in the distance; *step length(cm)*, walking distance (10 m) divided by the number of steps; *cadence(steps/min)*, the number of vertical lower trunk acceleration peaks divided by the walking duration of each trial; *step timing variability*, SDs between successive gait cycles over an entire walking trial. Gait cycles were determined by the vertical lower trunk acceleration peaks.

#### Walking stability variables

Each variable in this part was calculated in the AP, ML, and VT axes. *Acceleration root mean square (RMS)*, the dispersion of the measured acceleration signal relative to zero; *Harmonic Ratio (HR)*, the ratio of even harmonics and odd harmonics of the measured acceleration signal, reflecting the gait smoothness and symmetry [20]; *Step regularity (SR1)*, the amplitude of the first peak in the acceleration autocorrelation signal; *Stride regularity (SR2)*, the amplitude of the second peak in the acceleration autocorrelation signal; *Gait symmetry*, the closeness of SR1/SR2 to 1.0 [21]; *Gait variability*, the width of the dominant peak in the power spectrum of the measured acceleration signal [14].

### Statistical analysis

All statistical analyses were performed using SPSS Release 22 (SPSS Inc., Chicago, IL, USA). All continuous variables were described with mean ± standard deviation. The normality test was performed using the Kolmogorov-Smirnov test and variables with positively skewed distributions were log_10_ transformed before inferential analysis. Walking stability variables were first adjusted to walking speed to remove the influence of gait speed [22]. One-way ANOVA was performed to test the differences of gait variables between BPPV patients and healthy controls, and that among three disease stages of BPPV by DHI scores.

### Classification model of BPPV patients

A machine-learning based model was built to differentiate BPPV patients from healthy controls and to classify the severity of BPPV patients into 3 subgroups of DHI.

#### Feature selection

To improve the performance of classification model, all the gait variables are used as feature selection set. We classified and labeled the whole feature set into 4 groups: healthy, mild BPPV, moderate BPPV, and severe BPPV. In case of over fitting, principal component analysis was conducted on the input feature set and 4 principal components were used as selected features for model training.

#### Model training

Support vector machine (SVM) with a linear kernel was used to build the model due to its good performance with high dimensional data, high signal to noise ratio [23], and it outperformed other machine learning algorithms, i.e. multi-layer perceptron and the k-nearest neighbors in training gait data [24].

#### Model validation

Repeated 5-fold cross-validation was performed to evaluate the model performance, meaning that the dataset was split into 5 subsets, where 4 subsets were used for training the model and the remaining subset was used as an independent validation set. This training and validation were repeated 5 times where each time a different independent validation set was used. The receiver operating characteristic (ROC) curve and area under the curve (AUC) were used to evaluate the model performance in each fold.

## Results

### Participant characteristics

Demographic characteristics of the 27 BPPV patients and 27 healthy subjects are shown in Table 1. The experimental groups were of similar age, weight, height, and gender ratio (*p* > 0.05).

**Table 1:** Mean value and standard deviation of subject characteristics

	BPPV (n = 27)	Controls(n = 27)	*p*-value
Age (year)	56.5 ± 13.1	56.1 ± 10.8	0.63
Gender	16 F + 11 M	21 F + 6 M	0.28
Weight (Kg)	63.5 ± 10.8	59.6 ± 8.0	0.11
Height (cm)	162.0 ± 6.7	161.2 ± 5.1	0.45

### Gait variables

Gait variables highlighted significant alteration in temporospatial characteristics and walking stability between healthy subjects and individuals with BPPV (Table 2). Compared to healthy subjects, BPPV patients walked more slowly with decreased cadences and shorter step lengths (*p* < 0.05) (Table 2). The RMS of BPPV patients were found generally decreased than that of healthy subjects, but the significant difference was found only in the VT axis of both head and lower trunk (*p* < 0.05). There was no significant difference in the ML axis of both head and lower trunk between healthy people and individuals with BPPV (*p* > 0.05) (Table 2). In AP axis, although RMS in the head of BPPV patients was significantly lower than that of healthy subjects (*p* < 0.05), there was no statistical significance in the lower trunk (*p* > 0.05) (Table 2). With regard to the HR, BPPV patients was generally lower than that of healthy subjects, and there was statistical significance in the ML axis of both head and trunk and the VT axis of the lower trunk (*p* < 0.05) (Table 2).

**Table 2:** Gait variables between BPPV patients and healthy subjects

Variables	BPPV	Controls	*P*-Value
Temporospatial			
** Walking Speed (m/s)**	**1.12 ± 0.15**	**1.20 ± 0.12**	**0.048**
** Step length (cm)**	**75.78 ± 8.43**	**78.84 ± 5.86**	**0.043**
** Cadence(steps/min)**	**111.17 ± 10.69**	**119.54 ± 8.03**	**0.002**
Step timing variability	0.018 ± 0.009	0.016 ± 0.006	0.38
RMS			
Head			
** VT**	**0.178 ± 0.05**	**0.212 ± 0.04**	**0.02**
ML	0.11 ± 0.04	0.13 ± 0.03	0.09
** AP**	**0.13 ± 0.04**	**0.17 ± 0.03**	**< 0.01**
Trunk			
** VT**	**0.18 ± 0.06**	**0.22 ± 0.05**	**0.02**
ML	0.11 ± 0.03	0.10 ± 0.02	0.57
AP	0.07 ± 0.03	0.09 ± 0.04	0.06
HR			
Head			
VT	2.66 ± 0.86	3.09 ± 0.88	0.50
** ML**	**2.18 ± 0.54**	**2.78 ± 0.66**	**0.04**
AP	2.07 ± 0.48	2.48 ± 0.63	0.15
Trunk			
** VT**	**2.54 ± 0.74**	**2.98 ± 0.61**	**< 0.01**
** ML**	**2.59 ± 0.72**	**2.97 ± 0.77**	**0.02**
AP	1.50 ± 0.55	1.74 ± 0.59	0.14

RMS refers to root mean square; HR refers to harmonic ratio; VT, ML, and AP refer to the vertical axis, mediolateral axis, and anteroposterior axis, respectively. Parameters with significant difference between BPPV patients and healthy controls are highlighted in bold

**Fig. 2:**
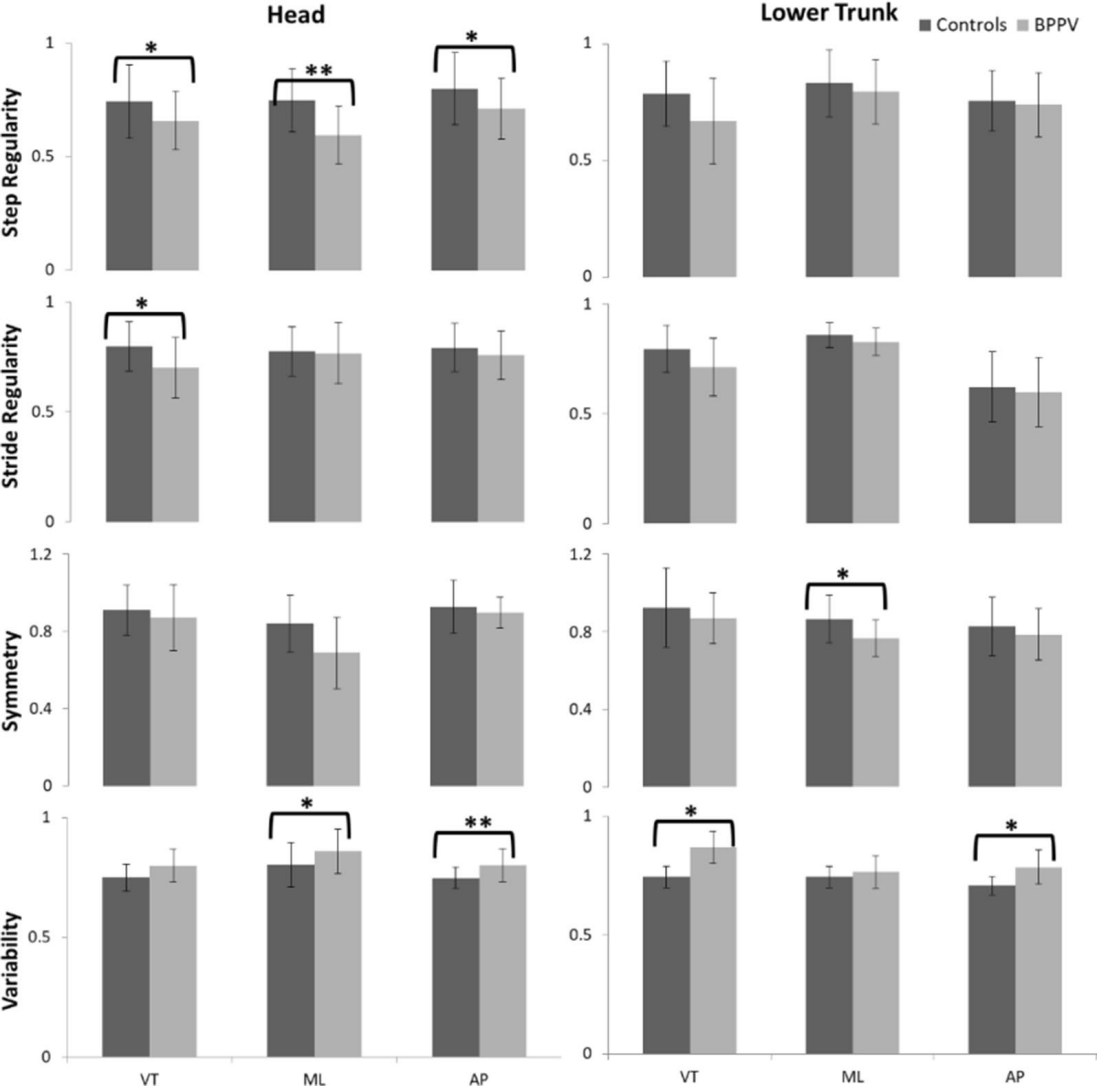
Differences in step regularity, stride regularity, gait symmetry and gait variability (* p < 0.05; ** p < 0.01). VT, ML, and AP refer to the vertical axis, mediolateral axis, and anteroposterior axis, respectively. Absolute value is adopted

When assessing the walking stability from the perspective of gait quality, decreased consistency of gait was found in BPPV patients, as detected in the lower step regularity in all three axes at the head (*p* < 0.05), lower stride regularity in the VT axis at the head (*p* < 0.05), reduced symmetry in the ML axis at the lower trunk (*p* < 0.05) (Fig. 2). The gait variability known as another marker for walking stability was found increased in BPPV patients in the ML and AP axes at the head, and in the VT and AP axes at the lower trunk, compared to healthy subjects (*p* < 0.05) (Fig. 2)

BPPV patients were assigned to the mild (DHI = 0–30), moderate (DHI = 31–60) and severe (DHI = 61–100) subgroups according to their DHI scores. Age, gender and temporospatial variables did not show significance differences among three DHI subgroups (p > 0.05) (Table 3). One-way ANOVA results for these variables found HR in the ML axis at the lower trunk and step regularity in the AP axis at the head showed significant differences among the three subgroups (*p* < 0.05) (Table 3).

**Table 3:** Demographics and gait variables among three DHI subgroups

Variables	Mild (N = 12) DHI 0–30	Moderate (N = 9) DHI 31–60	Severe (N = 6) DHI 61–100
Subject characteristics			
Age (year)	62 (39.75)	63 (47)	53.5 (49.25)
Gender	3 M + 9 F	5 M + 4 F	3 M + 3 F
Weight (Kg)	62 (59.25)	60 (56)	56.5 (52)
Height (cm)	161 (158.5)	160 (156)	159 (156.5)
Temporospatial variables			
Step length	73.18 (69.83)	80.57 (76.68)	75 (69.67)
Cadence	114.06 (107.53)	111.75 (104.07)	116.11 (96.48)
Walking speed	1.10 (1.01)	1.15 (1.06)	1.19 (0.97)
RMS			
Head, VT	0.16 (0.15)	0.17 (0.16)	0.21 (0.11)
Head, AP	0.13 (0.12)	0.14 (0.12)	0.11 (0.09)
Trunk, VT	0.18 (0.15)	0.17 (0.15)	0.19 (0.14)
HR			
Head, ML	1.86 (1.55)	2.00 (1.47)	1.60 (1.56)
Trunk, VT	2.31 (1.86)	2.13 (1.77)	2.09 (1.99)
Trunk, ML**	**2.50 (2.17)**	**2.40 (2.10)**	**1.76 (1.58)**
Step Regularity			
Head, VT	0.69 (0.65)	0.62 (0.53)	0.62 (0.57)
Head, ML	0.61 (0.55)	0.69 (0.48)	0.47 (0.23)
Head, AP*	**0.87 (0.81)**	**0.84 (0.75)**	**0.82 (0.76)**
Stride regularity			
Head, VT	0.78 (0.73)	0.80 (0.78)	0.76 (0.73)
Gait symmetry			
Trunk, ML	0.90 (0.84)	0.88 (0.86)	0.91 (0.74)
Gait variability			
Head, ML	0.77 (0.72)	0.85 (0.79)	0.81 (0.73)
Head, AP	0.79 (0.73)	0.78 (0.77)	0.80 (0.73)
Trunk, VT	0.76 (0.72)	0.79 (0.78)	0.76 (0.74)
Trunk, AP	0.77 (0.72)	0.78 (0.76)	0.81 (0.73)

DHI refers to DHI score. VT, ML, and AP refer to the vertical axis, mediolateral axis, and anteroposterior axis, respectively. * indicates p < 0.05. ** indicates p < 0.01. Parameters with significant difference between BPPV subgroups are highlighted in bold

### Classification model of BPPV disease

**Fig. 3:**
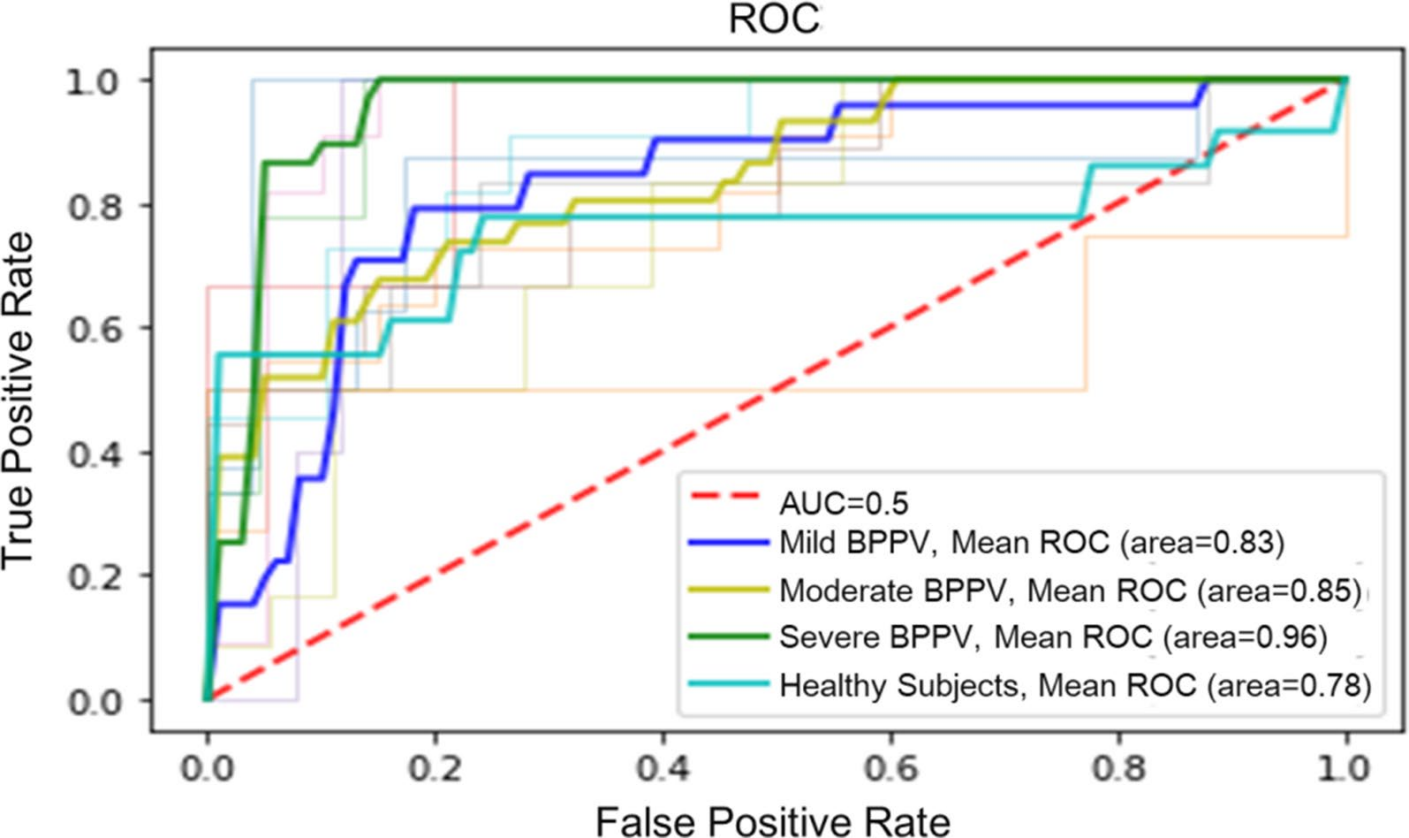
ROC and AUC for the classification of Healthy controls and DHI subgroups

The SVM model achieved AUCs of 0.83, 0.85 and 0.96 respectively for classifying the mild, moderate, severe stages of DHI subgroups and AUC of 0.78 for differentiating patients from healthy groups. The ROC curve is shown in Fig. 3.

## Discussion

To the best of our knowledge, this is the first study to analyze the walking stability of BPPV patients in clinical settings using wearable sensors. Results showed that patients with BPPV have significantly impaired walking stability even though a conservative gait was adopted. Furthermore, a SVM machine learning model based on all the gait variables automatically differentiated BPPV patients from healthy controls with average accuracy of 0.78 and classified the handicapping effects of dizziness imposed by BPPV disease according to DHI scores, with average accuracy of 0.83, 0.85, and 0.96 for mild, moderate, and severe subgroups, respectively.

In the current study, we found that BPPV patients exhibited significantly lower walking speed, step length, and cadence indicating their conservative gait during vertigo onset, which were consistent with previous findings [8, 9]. The conservative gait might be the compensation to the ineffective sensory organization and abnormal vestibulospinal output caused by impaired vestibular information and can be seen as a compensatory strategy to enhance the dynamic stability during walking and thus avoid falls [25].

The walking stability analysis of BPPV patients showed that the RMSs in ML axis of both head and trunk did not decrease significantly with slower gait speed. Previous studies have reported that instability during walking is primarily in the ML and decline in ML stability is a major risk factor of fall [26, 27]. The RMSs of acceleration in ML axis is often employed as an index to evaluate the walking stability and higher RMS is generally associated with higher postural disturbance and risk of falls [28, 29]. Thus, our findings reveal that patients with BPPV were not able to attenuate the ML and AP axes acceleration in a tolerable level to maintain a stable visual field and postural stability, which may explain the reason that BPPV patients still have high fall risks despite employing this more conservative strategy.

In the presented study, the lower HRs at the lower trunk in the VT and ML axis were found in BPPV patients while they walk at self-preferred speed. HRs has been used as a stability index to evaluate the smoothness of gait and higher HRs are interpreted as greater walking smoothness [20, 30]. Previous studies have applied the HRs from trunk acceleration data to assess the stability of the trajectory of the center of mass and investigated the balance control ability between older adults, individuals with Parkinson’s disease, and individuals with sensory impairment [31–33]. Consistence with previous studies, our findings suggest that the peripheral vestibular disorder in BPPV patients has affected their walking smoothness and balance control.

We also identified the alteration in the walking stability of BPPV patients from the perspective of gait quality. The significant lower step regularity, stride regularity and gait symmetry were found in BPPV patients, suggesting that patients are less able to regulate the repeated walking pace and to control the rhythmic displacements of the body during walking. Furthermore, we found significant higher gait variability in BPPV patients compared to healthy subjects, which was consistent with previous studies that patients with vestibular failure had increased variability during slow walking [34]. Gait variability has been investigated as a very important objective variable in differentiating patients with balance problems and increased gait variability were found strongly associated with higher risk of fall [35]. Thus, our finding demonstrates that the gait and balance disturbances are the main symptoms of BPPV patients which could be objectively assessed by sensor- based walking stability parameters.

DHI is a validated tool to evaluate the handicapping effects of dizziness in vestibular diseases. Previous studies have found that BPPV in general was associated with relatively higher DHI scores, indicating that BPPV patients are suffering from considerable dizziness handicap [36–38]. However, there was no association between dizziness handicap and the intensity of positional nystagmus during BPPV diagnostic maneuvers [38], and therefore there is still lack of an objective tool to diagnose the handicapping effects of BPPV disease. In this study, we found significant walking stability impairments shown by mediolateral HR at lower trunk and anteroposterior step regularity at head among mild, moderate and severe of DHI subgroups, while temporospatial parameters were no significant differences. These results proved our hypothesis that the gait disturbances imposed by the dizziness/vertigo in BPPV patients are mainly reflected in the balance function even if they adopt a conservative gait. Since DHI is a self-reported questionnaire to quantify the dizziness on a daily basis[36], we built a machine learning-based model to classify different DHI subgroups with good performance, providing an objective method for assessing and monitoring the handicapping effects of dizziness imposed by BPPV disease.

The Dix-Hallpike(DH) maneuver is the definitive test for BPPV diagnosis, however, the test procedure usually causes severe fright and discomfort in the patients. The diagnosis is mainly based on the experience of the physician, and the estimated sensitivity was 79 % while specificity was 75 % [4]. This may due to the patient’s transient nature and eye fatigue, and physicians may not observe the patient’s nystagmus during the test, leading to incorrect disease assessment. In this study, we developed a machine learning-based model to differentiate BPPV patients from healthy controls with an accuracy of 78 %, comparable to the golden diagnosis standard. Given that the wearable sensors are ease to use, portable and affordable, it is suggested that gait assessment with wearable technology and machine learning approach can be used as a simple screening test in BPPV diagnosis.

There were several limitations in this study. First, we only recruited the patients with posterior canal BPPV, thus the results may not be applied to the BPPV patients with other types (i.e. horizontal canal). Second, the subjects included in three DHI rating subgroups have a relatively small sample size. Third, we utilized SVM algorithm to classify the DHI rating groups of BPPV as a preliminary attempt to assess the disease severity and progression. A future research direction would be to investigate the posterior and lateral canal patients and to develop more efficient algorithms to assist in the diagnosis of BPPV disease.

## Conclusions

In conclusion, the study found that BPPV patients have impaired walking stability even though a more conservative gait is adopted. The wearable technology provides a promising way to assess the gait disturbances in BPPV disease in the clinical settings. Using the impaired walking stability characteristics of BPPV patients, a machine learning-based classification model can be used to differentiate patient from healthy controls and assess the handicapping effects of dizziness imposed by BPPV with promising performance. The study set the stage for future development of wearable technology in tracking of gait and balance disorders, and could serve to inform future interventions in BPPV disease.

## Data Availability

All code and data used for this study are available from the corresponding author on reasonable request.

## Abbreviations

BPPV: Benign paroxysmal positional vertigo
RMS: Root mean square
HR: Harmonic ratio
SVM: Support vector machine model
DHI: Dizziness handicap inventory
DH: Dix-Hallpike
AUC: Area under the curve
